# The Role of ^11^C-Choline-PET/CT-Guided Secondary Lymphadenectomy in Patients with PSA Failure after Radical Prostatectomy: Lessons Learned from Eight Cases

**DOI:** 10.1155/2012/601572

**Published:** 2011-07-31

**Authors:** Thomas Martini, Roman Mayr, Emanuela Trenti, Salvatore Palermo, Evi Comploj, Armin Pycha, Maria Zywica, Michele Lodde

**Affiliations:** ^1^Department of Urology, General Hospital of Bolzano, Lorenz Böhler Street 5, 39100 Bolzano, Italy; ^2^Department of Vascular Surgery, Bressanone Hospital, Dante Street 51, 39042 Bressanone, Italy

## Abstract

*Introduction*. ^11^C-choline-PET/CT is a promising technique for detection/restaging of patients with biochemical failure (BF) after curative therapy for prostate cancer (PCA). The aim of this paper was to evaluate the PSA response in patients with BF after radical prostatectomy (RP) who underwent secondary lymphadenectomy (LAD) due to ^11^C-choline-PET/CT findings. *Material and Methods*. Eight patients with BF and positive lymph nodes in ^11^C-choline-PET/CT after RP were retrospectively included in the study. Extended LAD until the common iliac arteries was performed in all patients. *Results*. Six of 8 patients had histologically proven lymph node metastases. Four patients showed an initial PSA reduction after LAD, and in 4 patients the PSA increased. Two of the latter had no histological lymph node metastases. *Conclusions*. Because 50% of our patients showed an initial PSA response, our data suggest that positive ^11^C-choline-PET/CT after RP and BF could help to select patients that could benefit from secondary LAD.

## 1. Introduction

The incidence of recurrent disease after the initial curative treatment of local PCA ranges from 30 to 50% after RP [1, 2] and up to 80% after extracorporal radiotherapy [3]. An increasing PSA is the most certain indicator for relapse, but it does not always help in differentiating between local recurrence and systemic spread of the disease [4]. PSA kinetics and imaging techniques play an important role in the diagnostic of PCA recurrence after primary treatment. 

Positron emission tomography/computed tomography with choline tracer (*¹¹*C-choline-PET/CT) has emerged as a promising technique for restaging patients with BF. Tumor cells are known to have a higher turnover of essential cell membrane components, such as phosphatidylcholine, [5]. After uptake by tumor cells, radioactive choline is phosphorylated in high concentration and built into the cell membrane and can be recognized by PET [6]. PCA or its metastases can be detected by *¹¹*C-choline-PET/CT [7]. The aim of this paper was to retrospectively evaluate the PSA response after secondary LAD in patients with a BF and positive lymph nodes in *¹¹*C-choline-PET/CT after RP.

## 2. Material and Methods

Eight consecutive patients between 2009 and 2011 with BF and positive lymph nodes in *¹¹*C-choline-PET/CT were retrospectively included in the study. Because of a PCA, 5 patients had initially undergone a retropubic RP with a pelvic LAD and 3 a perineal RP without LAD. Local recurrence was excluded by transrectal ultrasound and digital rectal examination. All patients were informed of the pure diagnostic value and the risk of a surgical intervention. *¹¹*C-choline-PET/CT imaging results were analysed by a nuclear medicine specialist in all 8 cases. The patients subsequently underwent a secondary open extended LAD, performed by 2 of our experienced department surgeons. The extended LAD consisted of the dissection of lymph nodes from the obturator fossa, the internal and external iliac artery, the paravesical lymph nodes, and the common iliac artery. In one patient, only the *¹¹*C-choline-PET/CT-positive lymph node around the external iliac artery was resected. Three to five weeks after the LAD, the PSA value was determined and then determined again at 3-month intervals.

## 3. Results

Patient characteristics are summarized in Tables 1 and 2. The median PSA value at the time of *¹¹*C-choline-PET/CT was 1.62 ng/mL (IQR 1.47 ng/mL–2.56 ng/mL). Definitive histological lymph node metastases could be found in 6 of 8 patients with positive *¹¹*C-choline-PET/CT. In 2 of the 6 patients with histological lymph node metastases, the localization was not concordant with the *¹¹*C-choline-PET/CT findings. Regarding PSA response after LAD, 4 patients showed a PSA increase: two of them showed no sign of histological lymph node metastases, and the other two had an increase of the PSA value, despite the resection of the positive nodes. Three of them underwent androgen deprivation therapy (ADT), and one patient has been under observation with a stable PSA (0.21 ng/mL) after LAD for the last 29 months. Out of the 4 patients with decreasing PSA, one patient had an undetectable PSA after 29 months of followup. A second patient showed a decrease in PSA from 2.43 ng/mL to 0.66 ng/mL and remained stable after 34 months of followup. After a period of 5 months maintaining initial PSA response, the third patient showed disease progression. He subsequently underwent an ADT, and 31 months later he became castration resistant. He then received chemotherapy with docetaxel. At 4 months of followup, the fourth patient has not yet shown any increase in PSA value.

## 4. Discussion

When BF occurs after a radical treatment for PCA, being able to differentiate between local recurrence and distant metastases plays a crucial role in choosing the appropriate course of treatment. The functional imaging modality PET/CT has been proven to be useful for restaging patients with a PSA value which increases after RP. However, differing figures can be found in the literature with regard to the sensitivity and specificity of this approach. 

De Jong et al. analyse the role of the *¹¹*C-choline-PET/CT for preoperative staging in 67 patients with histologically proven PCA. This work gave a sensitivity of 80%, a specificity of 96%, and an accuracy of 93% for the detection of lymph node metastases using *¹¹*C-choline-PET/CT. They conclude that this tracer has a higher sensitivity than standard CT and magnetic resonance imaging (MRI) [8].

In patients with BF after RP, Scattoni et al. reported a lower sensitivity 64% and a specificity of 90% for the *¹¹*C-choline-PET/CT for the detection of lymph node metastases [9].

The sensitivity of choline-PET/CT in patients with BF after radical treatment for PC could range between 38 and 98% as was recently reported by Picchio et al. in their review article. Such variability is to be attributed to differences among patient cohorts [10]. The type of tumor and primary treatment, extension of the LAD, and particularly the PSA value at the time of choline-PET/CT could influence the sensitivity and specificity of this imaging technique. Some authors have stated that no positive lymph nodes could be detected using *¹¹*C-choline-PET/CT in patients with BF and PSA level <5 ng/mL [11], but others have shown positive PET/CT findings in patients with PSA values <5 ng/mL, albeit with a sensitivity reduction [12], and indeed this is in concordant with our experience. For example, Picchio et al. concluded that the routine use of choline-PET/CT cannot be recommended for PSA values <1 ng/mL [10]. Castellucci et al. evaluated the role of *¹¹*C-choline-PET/CT in the restaging of 102 patients after RP with only a slight PSA increase <1.5 ng/mL during followup. *¹¹*C-choline-PET/CT showed positive findings in only 28% of the cases: local relapse in 7 patients, bone metastases in 13 patients, and lymph node metastases in 9 patients. PSA doubling time and initial node status were found to be significant and independent factors in a multivariate analysis for positive *¹¹*C-choline-PET/CT. PSA doubling time in patients with positive PET findings was 4.3 months and in PET-negative patients 13.3 months (*P* = 0.0001). They concluded that the optimal threshold for PSA doubling time was 7.2 months, providing *¹¹*C-choline- PET/CT 93% sensitivity and 74% specificity [13].

In our cohort, 2 patients with BF showed no signs of lymph node metastases in the final histological analysis but instead exhibited inflammatory altered tissue, and, in 2 further patients, no positive nodes where found were these were indicated by *¹¹*C-choline-PET/CT giving a positive predictive value (PPV) of 50%. Schilling et al. found a PPV of 70% in a retrospective work similar to ours [14]. Inflammatory node disease and small bowel activity (adherence and hernias) seem to imitate positive lymph nodes. This is a known phenomenon and can be explained by the high proliferation activity of intestinal mucosa [8]. Our study showed a PSA reduction in 4 patients (50%), and, in 3 cases (37.5%), this response persisted at 29, 34, and 4 months of followup in the respective patients.

Our results agree with the findings of Winter et al. In their study, 3 of 8 patients with single lymph node metastases reached a complete PSA remission without adjuvant therapy. They concluded that a selected patient group seems to benefit from secondary LAD [15]. Weckermann et al. also showed that especially low-risk patients profit from the resection of the lymph node metastases and that 60% of patients with resected metastases were free of relapse without adjuvant therapy after 18 months [16]. It should also be determined whether patients that benefit from a secondary LAD had an optimal LAD at the time of primary treatment. In fact, data in the literature affirm that microscopic metastatic pelvic node disease could be cured by means of surgery and extended LAD. Bader et al., for example, found that 38.5% of the patients with one positive lymph node, 10% with two, and 14% with multiple lymph node metastases remained relapse-free for 45 months in a cohort of post-RP and extended LAD patients [17].

## 5. Conclusions

Extended LAD should be carefully performed at the time of the first treatment. 

In case of BF without local recurrence, *¹¹*C-choline-PET/CT could be performed if the PSA value is >1 ng/mL, to select patients that may benefit from a secondary LAD. 

Since positive lymph nodes have been found outside the regions indicated by *¹¹*C-choline-PET/CT, a complete extended secondary LAD should always be performed.

## Figures and Tables

**Table 1:** 

Patient	Age	Primary treatment	Initial tumor stage	Gleason score	Hormonal therapy after primary treatment	Radiotherapy after primary treatment
1	57	RP	pT3aN0MxR1	9 (4 + 5)	+	+
2	54	RP	pT2cN0MxR0	8 (4 + 4)	+	+
3	62	RPP	pT2bNxMxR1	6 (3 + 3)	−	−
4	65	RP	pT3bN0MxR0	7 (4 + 3)	−	+
5	59	RP	pT2aN0MxR0	7 (4 + 3)	−	+
6	68	RPP	pT3aN0MxR1	6 (3 + 3)	+	+
7	68	RPP	pT3bN0MxR1	9 (5 + 4)	−	+
8	75	RP	pT4N1MxR1	7 (4 + 3)	−	+

RP: radical prostatectomy; RPP: Radical perineal prostatectomy.

**Table 2:** 

Patient	PSA 1 (ng/mL)	PSA 2 (ng/mL)	PSA 3 (ng/mL)	PSA 4 (ng/mL)	Lymph nodes resected *n*	Lymph nodes positive *n*
1	6.14	0	1.38	1.6	24	3
2	5.54	0.64	1.57	0.1	3	1
3	8.56	0	0.17	0.2	17	0
4	8.32	0	1.66	1.74	5	1
5	7.62	0	2.92	1.34	20	1
6	6.94	0.53	2.93	4.1	11	0
7	5.23	0	2.43	0.66	12	1
8	9.81	0.13	1.5	0.11	1	1

PSA 1: initial PSA at time of cancer diagnosis; PSA 2: PSA after primary treatment; PSA 3: PSA at time of PET imaging; PSA 4: PSA after lymphadenectomy.
